# A population‐based study of factors associated with systemic treatment in advanced prostate cancer decedents

**DOI:** 10.1002/cam4.5401

**Published:** 2022-11-17

**Authors:** Jennifer Leigh, Danial Qureshi, Ewa Sucha, Roshanak Mahdavi, Igal Kushnir, Luke T. Lavallée, Dominick Bosse, Colleen Webber, Peter Tanuseputro, Michael Ong

**Affiliations:** ^1^ Department of Medicine University of Ottawa Ottawa Ontario Canada; ^2^ Ottawa Hospital Research Institute Ottawa Ontario Canada; ^3^ Nuffield Department of Population Health University of Oxford England UK; ^4^ ICES University of Ottawa, Ottawa Hospital Research Institute Ottawa Ontario Canada; ^5^ Sackler Faculty of Medicine Tel Aviv University Tel Aviv Israel; ^6^ Division of Urology, Department of Surgery The Ottawa Hospital and University of Ottawa Ottawa Ontario Canada; ^7^ Division of Medical Oncology, Department of Medicine The Ottawa Hospital Ottawa Ontario Canada; ^8^ Bruyère Research Institute Ottawa Ontario Canada

**Keywords:** decedent, life‐prolonging therapy, prostate cancer, regional cancer center

## Abstract

**Introduction:**

Life‐prolonging therapies (LPTs) are rapidly evolving for the treatment of advanced prostate cancer, although factors associated with real‐world uptake are not well characterized.

**Methods:**

In this cohort of prostate‐cancer decedents, we analyzed factors associated with LPT access. Population‐level databases from Ontario, Canada identified patients 65 years or older with prostate cancer receiving androgen deprivation therapy and who died of prostate cancer between 2013 and 2017. Univariate and multivariable analyses assessed the association between baseline characteristics and receipt of LPT in the 2 years prior to death.

**Results:**

Of 3575 patients who died of prostate cancer, 40.4% (*n* = 1443) received LPT, which comprised abiraterone (66.3%), docetaxel (50.3%), enzalutamide (17.2%), radium‐223 (10.0%), and/or cabazitaxel (3.5%). Use of LPT increased by year of death (2013: 22.7%, 2014: 31.8%, 2015: 41.8%, 2016: 49.1%, and 2017: 57.9%, *p* < 0.0001), driven by uptake of all agents except docetaxel. Adjusted odds of use were higher for patients seen at Regional Cancer Centers (OR: 1.8, 95% CI: 1.5–2.1) and who received prior prostate‐directed therapy (OR: 1.3, 95% CI: 1.0–1.5), but lower with advanced age (≥85: OR: 0.54, 95% CI:0.39–0.75), increased chronic conditions (≥6: OR: 0.62, 95% CI: 0.43–0.92), and long‐term care residency (OR: 0.38, 95% CI: 0.17–0.89). Income, stage at presentation, and distance to the cancer center were not associated with LPT uptake.

**Conclusion:**

In this cohort of prostate cancer‐decedents, real‐world uptake of novel prostate cancer therapies occurred at substantially higher rates for patients receiving care at Regional Cancer Centers, reinforcing the potential benefits for treatment access for patients referred to specialist centers.

## Graphical abstract

Patients receiving care at Regional Cancer Centers were substantially more likely to receive advanced prostate cancer treatments that affect overall survival. In contrast, geography, income, and stage at presentation were not important factors. Physicians caring for patients with advanced prostate cancer should consider referral to specialized treatment centers.

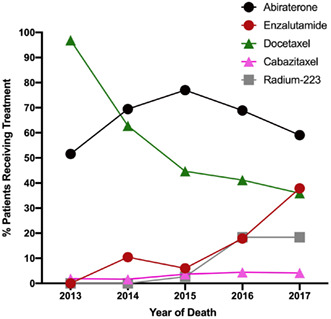

## 1 INTRODUCTION

Prostate cancer accounts for nearly 20% of all new cancer diagnoses in men.^1^ Although a majority are diagnosed at an early stage and with favorable prognosis,^1^, ^2^ a subset develops resistant disease following prostate‐directed treatments and subsequent androgen deprivation therapy (ADT). A further minority have aggressive behavior and present with “de novo” metastases at outset, requiring ADT for initial treatment. Together, these cohorts of ADT‐resistant prostate cancers account for 10% of all male cancer‐related deaths.^1^ Fortunately, for those with metastatic disease, systemic treatment has been rapidly evolving, with a number of ground‐breaking life‐prolonging therapies (LPT) that increase survival and quality of life of patients with metastatic castration‐resistant (mCRPC) and hormone‐sensitive prostate cancer.^3^, ^4^, ^5^, ^6^, ^7^, ^8^, ^9^

LPTs now approved for use in metastatic prostate cancer on the basis of improved survival, progression‐free, and symptomatic outcomes include docetaxel and cabazitaxel chemotherapy, androgen‐receptor axis therapies (ARATs), including abiraterone acetate and enzalutamide, and radiopharmaceuticals including radium‐223,^10^, ^11^ although the field continues to move rapidly with new treatments emerging. It is clear, however, from existing literature that approval of systemic treatments does not automatically lead to real‐world practice uptake.^12^ Frequently cited barriers include patient factors such as comorbidity, functional status, and increased age; provider belief in treatment efficacy and patient fitness; and logistical factors including the ability to consult a medical oncologist.^13^, ^14^, ^15^, ^16^, ^17^, ^18^, ^19^ For prostate cancer, the introduction of ARATs is expected to increase access to LPT given excellent survival outcomes, easier tolerability than chemotherapy, high quality of life data, and oral routes of administration.^10^, ^11^ Despite these characteristics, the real‐world uptake and barriers to access of ARATs and other LPTs are poorly understood.

In this study, we describe the use of LPT in Ontario prostate cancer decedents between 2013 and 2017, including factors associated with treatment receipt, and prescription trends over time. This time frame was chosen to encompass the periods after Health Canada approvals for abiraterone and enzalutamide in 2011 and 2013, respectively, as well as a time frame with data available for cause‐related mortality. We examine patient‐ and disease‐related factors associated with LPT receipt with the objective of understanding whether real‐world use of LPT is influenced by traditional barriers such as geographical distance of care, socioeconomic status, age, and comorbidity.

## 2 METHODS

### 2.1 Identification of decedents

We conducted a retrospective cohort study using population‐based administrative databases held at ICES (formerly the Institute for Clinical Evaluative Sciences) to identify all patients 65 years or older at the study index date in Ontario, Canada with a diagnosis of prostate cancer, who received ADT or had a bilateral orchiectomy after diagnosis and before the index date, and who died between 2013 and 2017 of prostate cancer. The study index date was defined as the date 2 years prior to the patient's death. A 2‐year look‐back period prior to death and therefore from the study index date was analyzed to determine LPT receipt in relation to patient and disease factors. The age 65 cutoff was selected as that is when patients become eligible for Ontario Drug Benefit (ODB), meaning there would be no funding restriction for access to therapy. The year 2017 was selected for data cutoff as at the time of this study cause of death data was not available past 2017. We used the STROBE cohort checklist when writing our report.^20^

### 2.2 Data sources

These datasets were linked using unique coded identifiers and analyzed at ICES. The databases utilized and descriptions of the information they provided can be found in Table S1. Use of the data in this project is authorized under section 45 of Ontario's Personal Health Information Protection Act (PHIPA) and does not require review by a Research Ethics Board.

### 2.3 Characteristics of interest

Patient characteristics including age, area‐level income quintile, Rurality Index of Ontario (RIO), count of chronic disease, Charlson Comorbidity Index Score (CCI), home care registration, straight‐line distance to the cancer center, and long‐term care (LTC) residency were collected using previously validated methods at study index data.^21^, ^22^, ^23^, ^24^, ^25^, ^26^, ^27^, ^28^ Physician involvement, number of visits by specialty, Regional Cancer Center registration, primary care rostering, and LPT receipt were all continuous variables that were examined within the 2 years between study index date and death. Receipt of radiotherapy or prostatectomy was examined at any time between diagnosis and death. The type of oncologist involved in care and the number of visits to oncologic specialists was identified using physician billing data from the Ontario Health Insurance Plan Database (OHIP). Rostering to a primary care physician was determined using the Client Agency Program Enrolment database. TNM stage and year of diagnosis were captured from the Ontario Cancer Registry, and prostate specific antigen at ADT initiation was captured from the Ontario Laboratories Information System. The drug codes utilized can be found in Tables S1 and S2.

### 2.4 Outcomes of interest

The primary outcome was the receipt of LPT in the last 2 years of life. Agents considered to be LPT were abiraterone, enzalutamide, docetaxel, cabazitaxel, and radium‐223, and their use within the look‐back period was captured using the ODB and New Drug Funding Program databases. A patient was considered to have received LPT if they had at least one prescription for any of the above‐listed drugs. A secondary outcome was to examine prescribing trends over time of each agent and determine whether certain agents are prescribed more frequently than others.

### 2.5 Statistical analysis

Descriptive statistics were used to describe patient and disease characteristics as categorized by LPT receipt. Categorical variables are reported as the number (*n*) and proportion (%) of patients, and continuous variables are reported as mean and standard deviation (SD). Factors associated with LPT receipt were examined using univariate and multivariable logistic regression models. Variables adjusted for included age, area‐level income quintile, distance to cancer center, count of chronic diseases, involvement of a medical, radiation, or uro oncologist, regional cancer center registration, LTC residency, stage at diagnosis, year of diagnosis, and prior prostate‐directed therapy. Statistical significance was defined as *p* ≤ 0.05. All analyses were conducted using SAS Enterprise Guide 7.1 (SAS Institute Inc.).

## 3 RESULTS

### 3.1 Receipt of therapy

There were 1443 of 3575 patients (40.4%) who received LPT during the lookback period. Type of LPT received included abiraterone (*n* = 957, 66.3%), docetaxel (*n* = 726, 50.3%), enzalutamide (*n* = 248, 17.2%), radium‐223 (*n* = 145, 10.0%), and cabazitaxel (*n* = 50, 3.5%). There were 1157 of these patients (80.2%) registered at a Regional Cancer Center. Prostate‐directed therapy was received by 918 (25.7%) patients overall, with 759 (21.2%) receiving prostate radiotherapy, 270 (7.6%) who underwent prostatectomy, and 111 (3.1%) who received both. There were 1746 patients (48.8%) who received palliative radiotherapy to the bone, and 313 (8.8%) in other regions.

### 3.2 Cohort characteristics

A total of 3575 prostate cancer patients who received ADT were identified to have prostate cancer‐related death between 2013 and 2017 (Table 1). In the lowest age bracket of 65–69, 143 (58.9%) received LPT, compared with 221 (22.3%) receiving LPT who were 85 years or older. The proportion receiving LPT was similar for patients living in the most urban centers (*n* = 905, 37.8%) and most rural (*n* = 17, 40.5%), and for those in the highest (*n* = 295, 41.2%) and lowest (*n* = 264, 38.4%) income brackets. The mean distance to the cancer center was 38.1 km for those who received LPT, and 35.4 km for patients that did not. There were 532 (14.9%) patients who had a CCI of ≤2, of whom 163 (30.6%) received LPT and 2389 (66.8%) who had a CCI of ≥5, of whom 1064 (44.5%) received LPT. A minority were long‐term care residents (*n* = 90, 2.52%), with 7 (7.8%) receiving LPT, and/or home care recipients (*n* = 593, 16.6%), with 202 (34.1%) receiving LPT.

**TABLE 1:** Patient characteristics of prostate cancer decedents. Patient characteristics are stratified by whether life‐prolonging therapy was received

Characteristic	Description	No Life‐Prolonging Therapy n (%) (*n* = 2132, 59.6%)	Life‐Prolonging Therapy n (%) (*n* = 1443, 40.4%)	Overall (*n* = 3575)
Age	65–69	100 (41.2)	143 (58.9)	243
	70–74	269 (41.3)	383 (58.7)	652
	75–79	406 (53.5)	353 (46.5)	759
	80–84	585 (63.0)	343 (37.0)	928
	85+	772 (77.7)	221 (22.3)	993
Area‐level income quintile	1 (lowest)	424 (61.6)	264 (38.4)	688
	2	441 (61.0)	282 (39.0)	723
	3	423 (58.6)	299 (41.4)	722
	4	416 (58.1)	300 (41.9)	716
	5 (highest)	420 (58.7)	295 (41.2)	715
Rurality Index for Ontario	0 to 9 (most urban)	1328 (59.5)	905 (37.8)	2233
	10 to 30	399 (60.6)	259 (40.5)	658
	31 to 45	230 (58.7)	162 (41.3)	392
	46 to 55	55 (56.7)	42 (43.3)	97
	56 to 75	72 (62.1)	44 (37.9)	116
	76 to 100 (least urban)	25 (59.5)	17 (40.5)	42
Distance to Cancer Center (km)	Mean (SD)	35.4 (56.2)	38.1 (55.6)	36.8 (55.9)
Charlson Comorbidity Index Score	≤2	369 (69.4)	163 (30.6)	532
	3–4	178 (78.8)	48 (21.2)	226
	≥5	1325 (55.5)	1064 (44.5)	2389
Count of Chronic Diseases	Mean (SD)	3.49 (1.94)	3.09 (1.76)	3.33 (1.88)
Physicians involved in their cancer care	Medical Oncologist	1006 (43.5)	1306 (56.5)	2312
	Radiation Oncologist	1220 (51.7)	1140 (48.3)	2360
	Urologist	1793 (60.9)	1152 (39.1)	2945
Mean number of visits by physician specialty (SD)	Medical Oncologist	9.67 (15.2)	27.9 (26.9)	20.0 (24.3)
	Radiation Oncologist	5.42 (5.02)	6.83 (6.00)	6.09 (5.56)
	Urologist	9.91 (9.22)	10.2 (7.97)	10.0 (8.75)
	Family Physician	10.8 (19.3)	12.9 (21.9)	11.7 (20.4)
Rostered to primary care	Yes	1759 (58.7)	1238 (41.3)	2997
	No	373 (64.5)	205 (35.5)	578
Consultation at regional cancer center	Yes	1146 (49.8)	1157 (50.2)	2303
	No	986 (77.5)	286 (22.5)	1272
Long‐Term Care Resident	Yes	83 (92.2)	7 (7.78)	90
	No	2049 (58.8)	1436 (41.2)	3485
Home care involvement	Yes	391 (65.9)	202 (34.1)	593
	No	1741 (58.3)	1241 (41.6)	2982

Provider care included a medical oncologist (*n* = 2312, 64.6%), radiation oncologist (*n* = 2360, 66.0%), and urologist (*n* = 2945, 82.4%), and LPT use was 56.5%, 48.3%, and 39.1%, respectively. For patients receiving LPT, the mean number of encounters were 27.9 medical oncology, 6.83 radiation oncology, and 10.2 urology visits. For patients not receiving LPT, mean number of encounters were 9.67 medical oncology, 5.42 radiation oncology, and 9.91 urology visits. Nine hundred and ninety‐seven (83.8%) were rostered to primary care, of which 1238 (41.3%) received LPT. Among 578 (16.2%) not rostered to primary care, 205 (35.5%) received LPT. Among 2303 (64.4%) patients registered at a Regional Cancer Center, 1157 (50.2%) received LPT. Among 1272 (35.6%) not registered, 286 (22.5%) received LPT.

Disease‐specific characteristics are outlined in Table 2. There were 909 patients (25.4%) diagnosed between 2002 and 2006, 1215 (34.0%) between 2007 and 2011, and 1451 (40.6%) between 2012 and 2016. LPT use by initial AJCC 6th edition stage: stage I/II/III 985 (27.5%), stage IV 1311 (36.7%), and stage missing 1279 (35.8%). LPT use in those with prostate‐directed therapy was 500/918 (54.5%) and 943/2657 (35.5%) in those who did not receive prostate‐directed therapy.

**TABLE 2:** Disease characteristics of prostate cancer decedents. Patient characteristics are stratified by whether life‐prolonging therapy was received. Stage at diagnosis and M status definitions are based on AJCC 6th edition definitions

Characteristic	Description	No Life‐Prolonging therapy *n* (%) (*n* = 2132)	Life‐Prolonging therapy n (%) (*n* = 1443)	Overall (*n* = 3575)
Stage at diagnosis	I/II/III	568 (57.7)	417 (42.3)	985
	IV	770 (58.7)	541 (41.3)	1311
	Missing	794 (62.1)	485 (37.9)	1279
M category at diagnosis	M0	483 (59.9)	324 (40.1)	483
	M1a	24 (57.1)	18 (42.9)	42
	M1b	536 (60.4)	351 (39.6)	351
	M1c	87 (63.0)	51 (37.0)	51
	Missing	1002 (58.9)	699 (41.1)	699
Year of diagnosis	2002–2006	534 (58.7)	375 (41.3)	909
	2007–2011	664 (54.6)	551 (45.4)	1215
	2012–2016	934 (64.4)	517 (35.6)	1451
PSA at first ADT initiation (ng/ml)	<10	116 (49.4)	119 (50.7)	235
	11–19	85 (53.5)	74 (46.5)	159
	20–99	240 (55.6)	192 (44.4)	432
	100–1000	233 (59.1)	161 (40.9)	394
	>1000	74 (61.7)	46 (38.3)	120
	Missing	1384 (61.9)	851 (38.1)	2235
Prostatectomy prior to death	Yes	93 (34.4)	177 (65.6)	270
	No	2039 (61.7)	1266 (38.3)	3305
Radiotherapy to the prostate prior to death	Yes	359 (47.3)	400 (52.7)	759
	No	1773 (61.7)	1043 (38.3)	2816
Prostatectomy or radiotherapy to prostate prior to death	Yes	418 (45.5)	500 (54.5)	918
	No	1714 (64.5)	943 (35.5)	2657
Radiotherapy to bone prior to death	Yes	823 (47.1)	923 (52.9)	1746
	No	1309 (71.6)	520 (28.4)	1829
Radiotherapy to other body parts	Yes	149 (47.6)	164 (52.4)	313
	No	1983 (60.8)	1279 (39.2)	3262

### 3.3 Univariate and multivariable relationships between LPT receipt and population characteristics

Univariate analysis revealed patients had higher odds of LPT use if they had Regional Cancer Center registration (OR 3.5 [95% CI 3.0–4.1]), receipt of prostate‐directed therapy (OR 2.2 [95% CI 1.9–2.5]), radiation oncologist involvement (OR 2.8 [95% CI 2.4–3.3]), and medical oncologist involvement (OR 11 [95% CI 8.8–12], Table 3). Odds of LPT use were higher in younger age groups compared with those 85 years or older (65–69: OR 5.0 [95% CI 3.7–6.7], 70–74: OR 5.0 [95% CI 4.0–6.2], 75–79: OR 3.0 [95% CI 2.5–3.7], 80–84: OR 2.0 [95%CI 1.7–2.5]). Odds of LPT use decreased in patients with ≥3 chronic conditions (3–5: OR 0.69 [95% CI 0.51–0.94], ≥6: OR 0.42 [95% CI 0.30–0.61]), and long‐term care residency (OR 0.12 [95% CI 0.055–0.26]). There were no differences in odds of LPT use with income quintile, rurality index, distance to the nearest cancer center, or TNM stage at diagnosis.

**TABLE 3:** Univariate and multivariable analyses of population characteristics and association with life‐prolonging therapy use. Univariate and multivariable analyses for the receipt for therapy

Characteristics		Univariate Odds ratio (95% CI)	Univariate p value	Multivariable Odds ratio (95% CI)	Multivariable value
Age	65–69	5.0 (3.7–6.7)	<0.0001	Reference	
	70–74	5.0 (4.0–6.2)	<0.0001	1.3 (0.93–1.8)	0.13
	75–79	3.0 (2.5–3.7)	<0.0001	0.91 (0.66–1.2)	0.56
	80–84	2.0 (1.7–2.5)	<0.0001	0.73 (0.53–1.0)	0.055
	85+	Reference		0.54 (0.39–0.75)	0.0003
Income	Quartile 1 (Lowest)	0.89 (0.72–1.1)	0.27	0.88 (0.69–1.1)	0.32
	Quartile 2	0.91 (0.74–1.1)	0.38	0.91 (0.71–1.2)	0.42
	Quartile 3	1.0 (0.82–1.2)	0.95	1.0 (0.80–1.3)	0.85
	Quartile 4	1.0 (0.83–1.3)	0.81	0.97 (0.76–1.24)	0.81
	Quartile 5 (Highest)	Reference		Reference	
Count of Chronic Diseases	0	Reference		Reference	
	1–2	0.80 (0.58–1.1)	0.17	0.88 (0.61–1.3)	0.49
	3–5	0.69 (0.51–0.94)	0.019	0.86 (0.60–1.2)	0.43
	6+	0.42 (0.30–0.61)	<0.0001	0.62 (0.41–0.94)	0.024
Physician Involved in their cancer care	Medical Oncologist	11 (8.8–12)	<0.0001	7.6 (6.2–9.3)	<0.0001
	Radiation Oncologist	2.8 (2.4–3.3)	<0.0001	1.2 (0.98–1.5)	0.067
	Urologist	0.75 (0.63–0.89)	0.001	0.84 (0.68–1.0)	0.10
Consultation at Regional Cancer Center	Yes	3.5 (3.0–4.1)	<0.0001	1.8 (1.5–2.1)	<0.0001
	No	Reference		Reference	
Distance to Cancer Center (km)	Mean	1.0 (1.0–1.0)	0.27	1.0 (0.99–1.0)	0.16
Long‐Term Care Resident	Yes	0.12 (0.055–0.26)	<0.0001	0.39 (0.17–0.89)	0.026
	No	Reference		Reference	
Stage at Diagnosis	Stage I/II/III	Reference		Reference	
	Stage IV	0.96 (0.81–1.3)	0.61	0.94 (0.76–1.2)	0.62
Prostatectomy or Radiotherapy to the prostate prior to death	Yes	2.2 (1.9–2.5)	<0.0001	1.3 (1.0–1.5)	0.0011
	No	Reference		Reference	

Multivariable analysis revealed higher odds of LPT use for patients with Regional Cancer Center registration (OR 1.8 [95% CI 1.5–2.1]) and receipt of prostate‐directed therapy (OR 1.3 [95% CI 1.0–1.5], Table 3). Lower odds of LPT use were observed in those 85 or older (OR 0.54 [0.39–0.75]), a higher number of co‐morbidities (≥6: OR 0.62 [95% CI 0.41–0.94]), and long‐term care residency (OR 0.39 [95% CI 0.17–0.89]). Odds of LPT use were not associated with distance to the nearest cancer center, income‐level quintile, and TMN stage at diagnosis.

### 3.4 Prescribing trends over time

**FIGURE 1:**
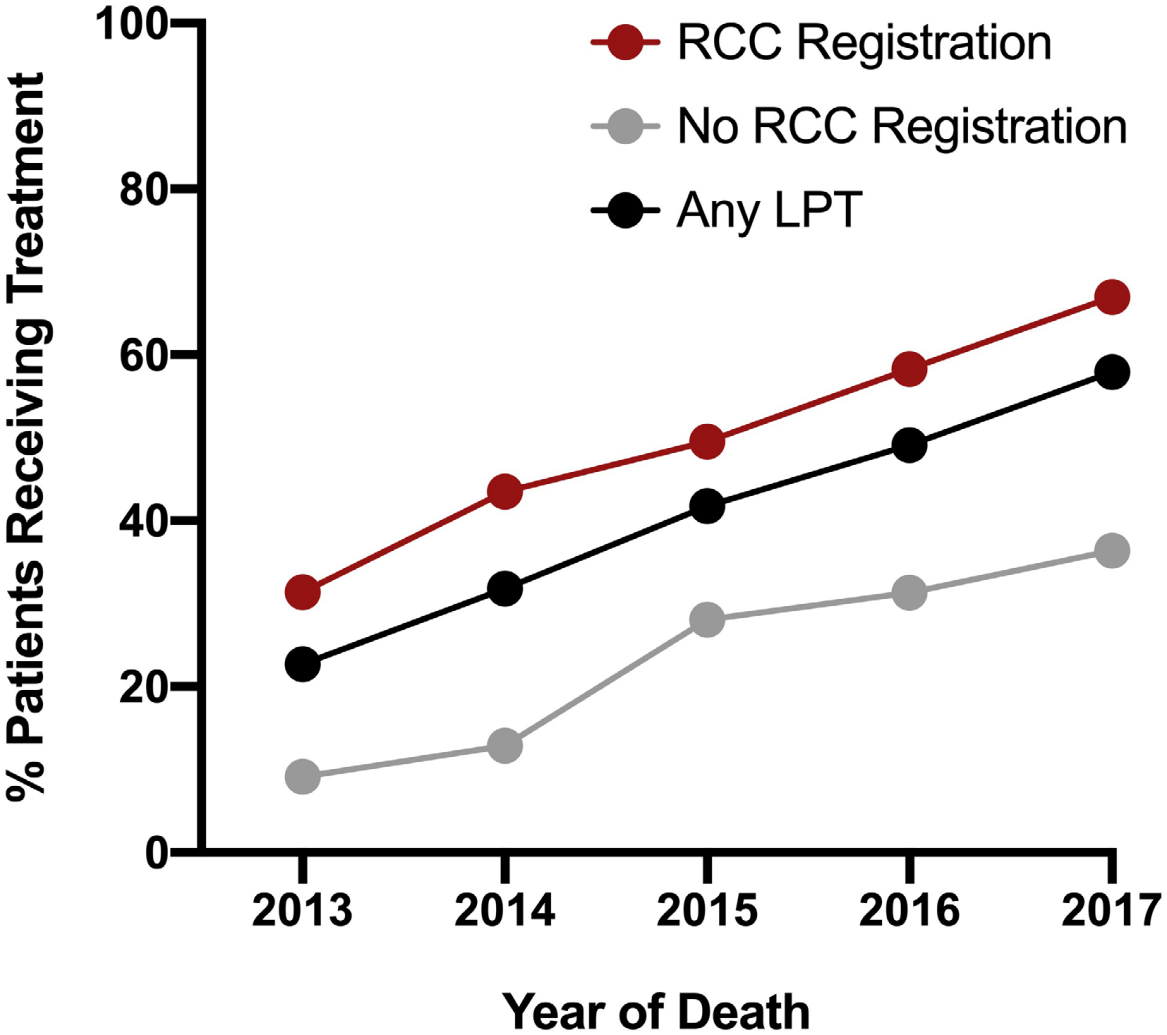
Proportion of patients receiving any Life‐Prolonging Therapy (LPT) prior to their death. Represented as % of all patients in our cohort who received any LPT prior to their death (black), % of patients who were registered at a Regional Cancer Center and received any LPT (red), and % of patients whose care occurred outside a Regional Cancer Center and received LPT (gray). Data are stratified by year of death.

**FIGURE 2:**
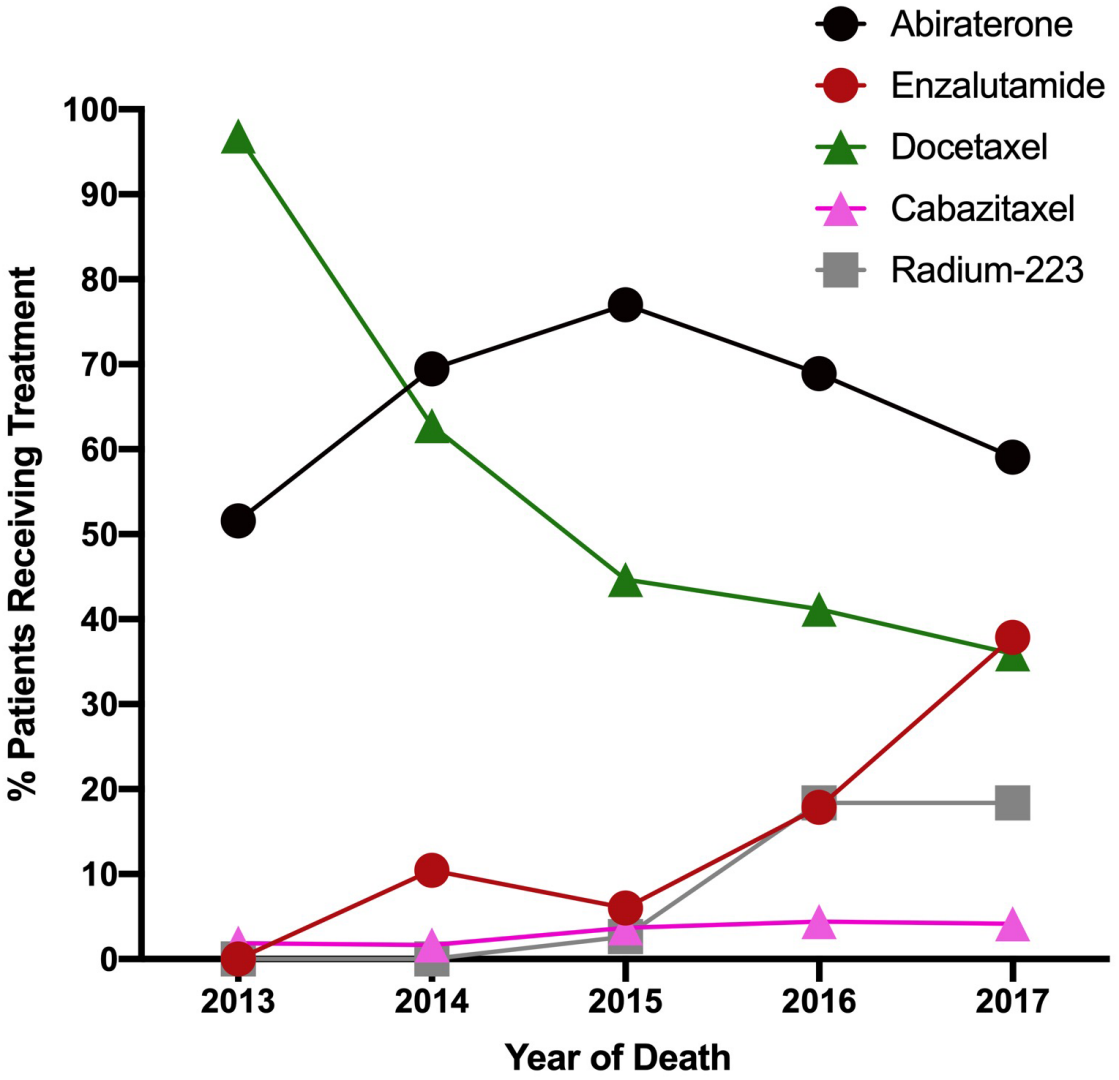
Uptake of Life‐Prolonging Therapy (LPT) is driven by novel agents. Represented as the percentage of receipt of each therapy for all patients in the cohort that received any type of LPT prior to death, stratified by year of death. Those who did not receive LPT were excluded. The following agents are included: abiraterone (black circle), enzalutamide (red circle), docetaxel (green triangle), cabazitaxel (pink triangle), and radium‐223 (gray square).

**FIGURE 3:**
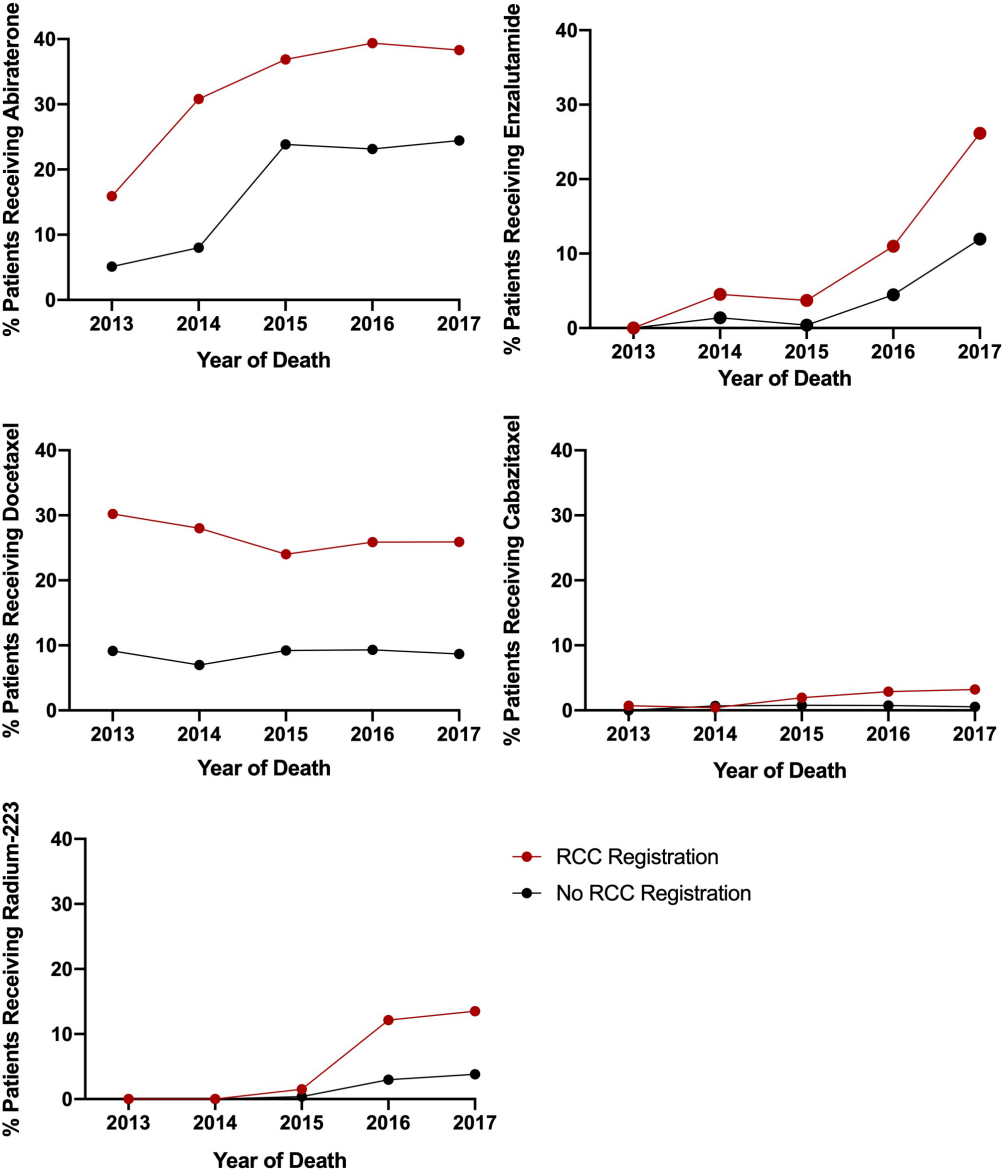
Uptake of life‐prolonging therapy (LPT) at Regional Cancer Centers is brisker and to a greater percentage of patients than at non‐Regional Cancer Center sites. This represents the percentage of all patients in the cohort who received each type of LPT, stratified by year of death. The types of LPT included are abiraterone (top left), enzalutamide (top right), docetaxel (middle left), cabazitaxel (middle right), and radium‐223 (bottom left). The red circle represents uptake for patients who were registered for care at a Regional Cancer Center, and the black circle represent uptake for patients who were not registered at a Regional Cancer Center.

The proportion of patients receiving LPT significantly increased by year of death (2013:22.7%, 2014:31.8%, 2015:41.8%, 2016:49.1%, 2017:57.9%, *p* < 0.0001, Figure 1). Of patients who received LPT, the use of abiraterone (*p* < 0.0001), enzalutamide (*p* < 0.0001), cabazitaxel (*p* < 0.0001), and radium‐223 (*p* < 0.0001) each increased in utilization by year of death, whereas docetaxel prescriptions were largely unchanged (Figures 2 and 3).

There were 2303 patients (64.4%) registered at a Regional Cancer Center. Registration increased by year of death (2013: 61.0%; 2014: 61.8%; 2015: 63.8%; 2016: 65.9%; and 2017: 70.3%, p = 0.0001). Uptake of LPT increased over time for registered patients (2013: 31.4%; 2014: 43.5%; 2015: 49.6%; 2016: 58.3%; and 2017: 67.0%, *p* < 0.0001, Figure 1), and for patients not registered at a Regional Cancer Center (2013: 9.2%; 2014: 12.9%; 2015: 28.1%; 2016: 31.3%; and 2017: 36.4%; *p* < 0.0001). Figure 3 outlines the uptake of each agent by Regional Cancer Center registration.

## 4 DISCUSSION

We demonstrate in a large population database of prostate cancer decedents after ADT treatment that a majority (59.6%) do not receive additional LPT in the 2 years preceding death. Income and rurality were not associated with LPT receipt, whereas those who were registered at Regional Cancer Centers, received prostate‐directed therapy, or were younger, less comorbid, and did not register at long‐term care had higher use of LPT. We also observe substantial increases in LPT use over time, which is largely driven by ARATs and to a lesser degree radium‐223. Although these trends were influenced by the timing of Health Canada and provincial approvals, ARATs that were available “post‐docetaxel” initially and then “pre‐docetaxel” in 2014–2015 had vastly higher uptake compared with cabazitaxel despite similar availability, with its approval postdocetaxel in 2011.^29^, ^30^, ^31^, ^32^, ^33^ Evolving indications for ARATs and docetaxel for metastatic hormone‐sensitive disease will clearly impact the 40–50% of patients in this cohort who may have benefitted from earlier access to LPT.^34^, ^35^, ^36^, ^37^, ^38^

Regional Cancer Center registration was a major factor associated with LPT use, which is consistent with prior data suggesting that specialist cancer center care leads to improved outcomes and therapy access.^39^ Specialist centers generally have greater drug familiarity through early access in clinical trials, specialized practices, and drug navigation services that promote the ability to access novel LPT.^40^ In our cohort, uptake of ARATs was brisk whether or not patients were registered at a Regional Cancer Center, whereas it was substantially higher at Regional Cancer Centers for radium‐223 and cabazitaxel, reflecting a larger capacity for the introduction of these specialized therapies. Additionally, uptake of all drugs occurred at higher rates overall at Regional Cancer Centers. This suggests that knowledge translation of clinical trial findings occurs at a fast pace at Regional Cancer Centers, which is a strength that can be potentially utilized to assist with quicker adoption of these therapies across the province through tools like educational sessions. Prior prostate‐directed therapy was also associated with increased LPT use. Patients with initially localized disease have improved prognoses compared to those with de novo metastatic disease, and therefore have a longer window to receive LPT. Prostate radiotherapy may also confer a survival advantage for low‐volume metastatic disease.^34^, ^35^, ^36^ The small proportion of patients who received localized treatment in our cohort speaks to the highly controllable state of most localized prostate cancer and the importance of identifying potentially lethal diseases within this population.

Factors negatively associated with LPT use included older age, increasing co‐morbidities, and long‐term care registration. Patients with increasing age and comorbidity have been shown to receive less systemic therapy in cancer care.^18^, ^41^ The reasoning behind this is multifactorial, including greater risk of harm in frail patients, patient goals of care including increasing preferences placed on quality of life and minimizing toxicities, and lack of evidence generalizability as they are often excluded from trials.^41^ Although evidence suggests that ARATs are well tolerated in fit elderly patients and can promote or preserve the quality of life,^41^ our study still observes a downward trend for LPT use with increasing age. Other important factors in treatment decisions such as the social support elderly patients have could not be assessed. Interestingly, rurality, distance to cancer center, and income were not associated with LPT use. While the fact that free drug access in our cohort may have influenced the role that income played, it is widely understood that the impact of socioeconomic status is complex and has a larger influence on healthcare access than the ability to afford a medication alone. Existing literature focusing on the impact of these factors is heterogeneous, indicating that barriers to accessing cancer care are specific to both treatment modality and disease site.^15^, ^17^, ^18^

Study limitations include reliance on data available in the administrative databases, which lack details that play into the treatment decision‐making process including patient preferences, treatment indication, and line of therapy. Patients under 65 were excluded as they are not automatically covered under ODB for oral drugs. Additionally, receipt of any therapies discontinued for patients prior to the last 2 years of their life would not have been captured. Oncology drugs following Health Canada approval are also often initially funded by private insurance or drug company programs, which could not be captured. Last, we did not restrict our cohort to those with a formal definition of mCRPC as our primary objective was to identify marginalized patients who did not receive LPT, and marginalized patients would be less likely to fulfill formal definitions within the administrative data set. A sensitivity analysis in formally defined mCRPC was largely concordant (Tables S3 and S4). Future studies examining LPT receipt in formally defined mCRPC, younger patients, and immigrant patients will be important in obtaining a more thorough understanding of LPT usage in lethal prostate cancer.

In conclusion, we performed a population‐based study of prostate cancer decedents receiving androgen deprivation therapy and found that patients who are seen at specialist cancer centers, receive prostate‐directed treatment, are younger, less comorbid, and not long‐term care residents have higher odds of receiving LPT. Amongst these, a key modifiable factor is caring for patients at a Regional Cancer Center. We identified that substantial gains have been made in delivery over time, driven largely by the introduction of abiraterone, enzalutamide, and radium‐223. Despite this, a substantial proportion of patients do not yet access LPT. In this cohort with universal access to healthcare and drug benefit, there were no differences detected on the basis of income, remoteness, or rurality for use of LPT. Future directions for research and policy should consider models of care that improve patient access to specialist multidisciplinary cancer center consultation as well as education outreach from these centers as LPT continues to rapidly evolve to encompass more novel treatments and earlier disease states.

## AUTHOR CONTRIBUTIONS

**Michael Ong, Peter Tanuseputro, Jennifer Leigh:** Conceptualization (equal); data curation (equal); formal analysis (equal); writing – original draft (equal); **Dominick Bosse, Peter Tanuseputro, Igal Kushnir, Luke Lavallee, Danial Quereshi:** writing – review and editing (equal). **Ewa Sucha:** Formal analysis (equal). **Roshanak Mahdavi:** Formal analysis (equal).

## FUNDING INFORMATION

This work was supported by funding from the Genitourinary Medical Oncologists of Canada (GUMOC) Astellas Research Grant Program. This work was also supported by ICES (formerly the Institute for Clinical Evaluative Sciences), which is an independent, nonprofit research institute funded by an annual grant from the Ontario Ministry of Health (MOH) and the Ministry of Long‐Term Care (MLTC).

## CONFLICTS OF INTEREST

LTL is an advisory board participant for Sanofi, AbbVie, Janssen, Bayer, and Knight. DB has received an honorarium for advisory boards or speaker fees from Bayer, Janssen, Ipsen, Amgen, Pfizer, AstraZeneca, BMS, and AbbVie. MO has been a consultant to and received honoraria from Janssen and Bayer. All other authors have no conflicts to disclose.

## ETHICAL APPROVAL

Use of the data in this project is authorized under section 45 of Ontario's Personal Health Information Protection Act (PHIPA) and does not require review by a Research Ethics Board.

## Supporting information


Appendix S1

## Data Availability

The data that support the findings of this study are available from the corresponding author upon reasonable request.
