# Supplementary material for: A population‐based study of factors associated with systemic treatment in advanced prostate cancer decedents
Source: Cancer Med. 2022 Nov 17;12(5):5569–79. doi: 10.1002/cam4.5401 (PMC10028120; doi:10.1002/cam4.5401)
Supplement: Supplementary file 1 — Appendix S1 [file CAM4-12-5569-s001.docx]

**Supplementary Data**

**Table 1. Databases Utilized to Collect Study Variables.** This table outlines the databases hosted under ICES that were used to collect the data included in this study, accompanied by a brief description of information gathered from that database.

| **Database** | **Description** |
| --- | --- |
| Ontario Drug Benefit (ODB) | Captures all ODB drug claims by patients and was utilized to identify receipt of drugs funded by this program. |
| Ontario Cancer Registry (OCR) | Provincial database of information for all those diagnosed with cancer. Date and diagnosis of prostate cancer and year of death were captured utilizing this. |
| Discharge Abstract Database (DAD) | Captures all acute care use. This was utilized to help capture those who underwent bilateral orchiectomy, admission to a palliative care unit, and metastatic status within 6 months of diagnosis. |
| Registered Persons Database (RPDB) | Utilized to capture demographic data including age, sex, and postal code. |
| Ontario Health Insurance Plan Claims Database (OHIP) | Utilized to capture all claims for physician services, including physician involvement, procedures performed, and number of visits to them during the study period. |
| National Ambulatory Care Reporting System (NACRS) | Captured all provincial emergency department encounters. |
| Cancer Activity Level Reporting (ALR) | Captures activity within the cancer system such as treatment sessions and clinic visits, and was utilized to determine regional cancer center registration, and receipt of some systemic therapies and radiotherapy. |
| Client Agency Program Enrolment (CAPE) | Captures information on a patient’s association with a primary care physician or care team and was utilized to determine whether patients were rostered to primary care. |
| Continuing Care Reporting System (CCRS) | Captured whether a patient is a long-term care resident. |
| Ontario Census Area Profiles (CENSUS) | Captured rurality and income quintiles using patient postal codes. |
| Postal Code Conversion File (PCCF) | Linked six-character postal codes and standard census data for that geographic area. |
| ICES Physician Database (IPDB) | Utilized to capture physician specialty. |
| New Drug Funding Program (NDFP) | Captured cancer drugs administered in hospitals or cancer centers that are funded by this program. |
| Ontario Laboratories Information System (OLIS) | Captured laboratory data. |
| Home Care Database (HCD) | Captured whether any home care services were delivered to patients. |
| Information about Ontario health care institutions funded by the Ministry of Health and Long-Term Care (INST) | Captured data about all publicly funded healthcare institutions. |

**Table 2. Drug Identification Numbers (DIN) utilized in data extraction.** This table outlines the drug information numbers (DIN) that were used to determine whether a patient received a particular drug. The New Drug Funding Program (NDFP) database was also utilized to look for drug receipt, and is not represented here.

| **Drug Category** | **Drug Name** | **Drug Identification Number** |
| --- | --- | --- |
| Androgen Deprivation Therapy | Leuprolide | 00884502, 00836273, 02239834, 02230248, 02239833, 02248239, 02248240, 02248999, 02268892 |
|  | Goserelin | 02049325, 02225905 |
|  | Triptorelin | 09857199, 09857200, 02240000, 02243856, 02412322 |
|  | Degarelix | 02337037, 02337029 |
|  | Buserelin | 02225166, 02225158, 02228955, 02240749 |
|  | Lupron | 00727695 |
|  | Histrelin | 02278383 |
| Prostate Cancer Life Prolonging Therapy | Abiraterone | 02457113; 02371065 |
|  | Enzalutamide | 02407329; 99400867 |
|  | Docetaxel | Captured solely from NDFP |
|  | Cabazitaxel | Captured solely from NDFP |
|  | Radium-223 | Captured solely from NDFP |

**Table 3. Patient characteristics in prostate cancer decedents, stratified by CRPC status.** Patient characteristics of the cohort outlined by whether CRPC status was able to be confirmed and whether or not life prolonging therapy was received. Cells donated with a (*) are modified with ranges and percentages removed in order to protect patient identity.

| Characteristic | Description | CRPC Confirmed -No Life Prolonging Therapy  n (%)  (n=780, 45.6%) | CRPC Confirmed-Life Prolonging Therapy  n (%)  (n=930, 54.4%) | CRPC Not Confirmed-  No Life Prolonging Therapy  n (%)  (n=1352, 72.5%) | CRPC Not Confirmed- Life Prolonging Therapy  n (%)  (n=513, 27.5%) |
| --- | --- | --- | --- | --- | --- |
| Age | 65-69 | 42 (30.2) | 97 (69.8) | 58 (55.8) | 46 (44.2) |
|  | 70-74 | 93 (27.4) | 247 (72.6) | 176 (56.4) | 136 (43.6) |
|  | 75-79 | 137 (38.0) | 224 (62.0) | 269 (67.6) | 129 (32.4) |
|  | 80-84 | 211 (49.5) | 215 (50.5) | 374 (74.5) | 128 (25.5) |
|  | 85+ | 297 (66.9) | 147 (33.1) | 475 (86.5) | 74 (13.5) |
| Area Level income quintile | 1 (lowest) | 146 (45.8) | 173 (54.2) | 278 (75.3) | 91 (24.7) |
|  | 2 | 144 (44.4) | 180 (55.6) | 297 (74.4) | 102 (25.6) |
|  | 3 | 150 (43.9) | 192 (56.1) | 273 (71.8) | 107 (28.2) |
|  | 4 | 163 (46.6) | 187 (53.4) | 253 (69.1) | 113 (30.9) |
|  | 5 (highest) | 176 (47.4) | 195 (52.6) | 244 (70.9) | 100 (29.1) |
| Rurality Index for Ontario | 0 to 9 (most urban) | 520 (47.1) | 584 (52.9) | 808 (71.6) | 321 (28.4) |
|  | 10 to 30 | 147 (46.8) | 167 (53.2) | 252 (73.3) | 92 (26.7) |
|  | 31 to 45 | 71 (40.1) | 106 (59.9) | 159 (74.0) | 56 (26.0) |
|  | 46 to 55 | 18 (36.0) | 32 (64.0) | 37 (78.7) | 10 (21.3) |
|  | 56 to 100 (least urban) | 20 (37.0) | 34 (63.0) | 77 (74.0) | 27 (26.0) |
| Charlson Comorbidity Index Score | ≤2 | 131 (54.6) | 109 (45.4) | 238 (81.5) | 54 (18.5) |
|  | 3-4 | 52 (60.5) | 34 (39.5) | 126 (90.0) | 14 (10.0) |
|  | ≥5 | 492 (42.4) | 674 (57.8) | 833 (68.1) | 390 (31.9) |
| Count of Chronic Diseases | 0 | 25 (35.2) | 46 (64.8) | 63 (61.2) | 40 (38.8) |
|  | 1-2 | 214 (40.8) | 310 (59.2) | 399 (70.4) | 168 (29.6) |
|  | 3-5 | 413 (46.3) | 479 (53.7) | 691 (72.3) | 265 (27.7) |
|  | 5-10 | 128 (57.4) | 95 (42.6) | 196 (83.1) | 40 (16.9) |
| Physicians involved in their cancer care | Medical Oncologist | 462 (35.2) | 849 (64.8) | 544 (54.3) | 457 (45.7) |
|  | Radiation Oncologist | 467 (39.4) | 718 (60.6) | 753 (64.1) | 422 (35.9) |
|  | Urologist | 682 (48.2) | 733 (51.8) | 1111 (72.6) | 419 (27.4) |
| Rostered to Primary Care | Yes | 657 (44.9) | 805 (55.1) | 1102 (71.8) | 433 (28.2) |
|  | No | 123 (49.6) | 125 (50.4) | 250 (75.8) | 80 (24.2) |
| Consultation at Regional Cancer Center | Yes | 482 (38.5) | 770 (61.5) | 664 (63.2) | 387 (36.8) |
|  | No | 298 (65.1) | 160 (34.9) | 688(84.5) | 126 (15.5) |
| Long Term Care Resident | Yes | 21 | 1-5* | 62 | 1-5 * |
|  | No | 759 | 925-929* | 1290 | 508-512* |
| Home Care Involvement | Yes | 147 (52.5) | 133 (47.5) | 244 (78.0) | 69 (22.0) |
|  | No | 633 (44.3) | 797 (55.7) | 1108 (71.4) | 444 (28.6) |

**Table 4. Disease characteristics of prostate cancer decedents, stratified by CRPC status.** Patient characteristics are stratified by whether CRPC status was able to be confirmed and whether life prolonging therapy was received. Stage at diagnosis and M status definitions are based on AJCC 6^th^ edition definitions.

| Characteristic | Description | CRPC Confirmed- No Life Prolonging Therapy  n (%)  (n=780, 45.6%) | CRPC Confirmed-  Life Prolonging Therapy  n (%)  (n=930, 54.4%) | CRPC Not Confirmed -No Life Prolonging Therapy  n (%)  (n=1352, 72.5%) | CRPC Not Confirmed-  Life Prolonging Therapy  n (%)  (n=513, 27.5%) |
| --- | --- | --- | --- | --- | --- |
| Stage at Diagnosis | I/II/III | 192 (42.5) | 260 (57.5) | 335 (71.9) | 131 (28.1) |
|  | IV | 173 (42.0) | 239 (58.0) | 378 (68.0) | 178 (32.0) |
|  | Missing | 415 (49.1) | 431 (50.9) | 639 (75.8) | 204 (24.2) |
| M Category at Diagnosis | M0 | 174 (45.2) | 211 (54.8) | 309 (73.2) | 113 (26.8) |
|  | M1/M1a | 8 (42.1) | 11 (57.9) | 16 (69.6) | 7 (30.4) |
|  | M1b | 167 (44.8) | 206 (55.2) | 369 (71.8) | 145 (28.2) |
|  | M1c | 18 (47.4) | 20 (52.6) | 69 (69.0) | 31 (31.0) |
|  | Missing | 413 (46.1) | 482 (53.9) | 589 (73.1) | 217 (26.9) |
| Year of Diagnosis | 2002-2006 | 230 (45.4) | 276 (54.5) | 304 (75.4) | 99 (24.6) |
|  | 2007-2011 | 313 (44.3) | 393 (55.7) | 351 (69.0) | 158 (31.0) |
|  | 2012-2016 | 237 (47.6) | 261 (52.4) | 697 (73.1) | 256 (26.9) |
| PSA at First ADT Initiation (ng/mL) | <10 | 50 (37.0) | 85 (63.0) | 66 (66.0) | 34 (34.0) |
|  | 11-19 | 42 (40.8) | 61 (59.2) | 43 (76.8) | 13 (23.2) |
|  | 20-99 | 120 (45.1) | 146 (54.9) | 120 (72.3) | 46 (27.7) |
|  | 100-1000 | 98 (50.8) | 95 (49.2) | 135 (67.2) | 66 (32.8) |
|  | >1000 | 28 (47.5) | 31 (52.5) | 46 (75.4) | 15 (24.6) |
|  | Missing | 442 (46.3) | 512 (53.7) | 942 (73.5) | 339 (26.5) |
| Prostatectomy prior to death | Yes | 45 (24.5) | 139 (75.5) | 48 (55.8) | 38 (44.2) |
|  | No | 735 (48.2) | 791 (51.8) | 1304 (73.3) | 475 (26.7) |
| Radiotherapy to prostate prior to death | Yes | 137 (32.9) | 280 (67.1) | 222 (64.9) | 120 (35.1) |
|  | No | 643 (49.7) | 650 (50.3) | 1130 (74.2) | 393 (25.8) |
| Prostatectomy or radiotherapy to prostate prior to death | Yes | 162 (31.1) | 359 (68.9) | 256 (64.5) | 141 (35.5) |
|  | No | 618 (52.0) | 571 (48.0) | 1096 (74.7) | 372 (25.3) |
| Radiotherapy to bone prior to death | Yes | 328 (36.0) | 582 (64.0) | 495 (59.2) | 341 (40.8) |
|  | No | 452 (56.5) | 348 (43.5) | 857 (83.3) | 172 (16.7) |
| Radiotherapy to other body parts | Yes | 53 (33.8) | 104 (66.2) | 96 (61.5) | 60 (38.5) |
|  | No | 727 (46.8) | 826 (53.2) | 1256 (73.5) | 453 (26.5) |

**Table 5. Chronic Conditions Present in the Decedent Cohort.** The number of prostate cancer decedents with 18 common chronic conditions, stratified by the percentage of patients with that condition who received life prolonging therapy. The overall column represents the total number of patients and overall percentage in the cohort who had each condition.

| Chronic Condition | No Life Prolonging Therapy  n (%) | Life Prolonging Therapy  n (%) | Overall  (n=3575) |
| --- | --- | --- | --- |
| Acute Myocardial Infarction | 22 (64.7) | 12 (35.3) | 34 (1.0) |
| Arrythmia | 411 (64.0) | 231 (36.0) | 642 (18.0) |
| Asthma | 235 (64.6) | 129 (35.4) | 364 (10.2) |
| Cancer (non-prostate) | 639 (52.7) | 574 (47.3) | 1213 (33.9) |
| CHF | 366 (73.2) | 134 (26.8) | 500 (14.0) |
| COPD | 323 (73.2) | 118 (26.8) | 441 (12.3) |
| Chronic Coronary Artery Disease | 765 (62.0) | 468 (38.0) | 1233 (34.5) |
| Diabetes | 716 (62.4) | 432 (37.6) | 1148 (32.1) |
| Dementia | 272 (81.9) | 60 (18.1) | 332 (9.3) |
| Hypertension | 1668 (61.3) | 1053 (38.7) | 2721 (76.1) |
| Inflammatory Bowel Disease | 12 (52.2) | 11 (47.8) | 23 (0.6) |
| Non-psychotic Mood Disorder | 203 (57.3) | 151 (42.7) | 354 (9.9) |
| Other Mental Health Condition | 88 (66.2) | 45 (33.8) | 133 (3.7) |
| Osteoporosis | 75 (53.2) | 66 (46.8) | 141 (3.9) |
| Osteoarthritis | 1098 (60.4) | 721 (39.6) | 1819 (50.9) |
| Renal Disease | 315 (65.6) | 165 (34.4) | 480 (13.4) |
| Rheumatoid Arthritis | 29 (60.4) | 19 (39.6) | 48 (1.3) |
| Stroke | 200 (71.9) | 78 (28.1) | 278 (7.8) |

**Table 6. Receipt of Life Prolonging Therapy in Each Local Integrated Health Network.** The proportion of prostate cancer decedents receiving life prolonging therapy in each of Ontario’s fourteen Local Integrated Health Networks (LHIN). Toronto Central is used as the reference for comparison of receipt.

| Local Health Integration Network | No Life Prolonging Therapy  n (%)  (n=2132, 59.6%) | Life Prolonging Therapy  n (%)  (n=1443, 40.4%) | Overall  (n=3575) | Odds ratio (95% CI) | p value |
| --- | --- | --- | --- | --- | --- |
| Erie St Clair | 107 (61.8) | 66 (38.2) | 173 | 0.98 (0.66-1.5) | 0.91 |
| Southwest | 170 (56.5) | 131 (43.5) | 301 | 1.2 (0.88-1.7) | 0.24 |
| Waterloo Wellington | 121 (60.5) | 79 (39.5) | 200 | 1.0 (0.71-1.5) | 0.86 |
| Hamilton Niagara Haldimand Brant | 281 (62.4) | 169 (37.6) | 450 | 0.95 (0.7-1.3) | 0.76 |
| Central West | 87 (56.1) | 68 (43.9) | 155 | 1.2 (0.83-1.9) | 0.29 |
| Mississauga Halton | 157 (64.1) | 88 (35.9) | 245 | 0.89 (0.62-1.3) | 0.52 |
| Toronto Central | 168 (61.3) | 106 (38.7) | 274 | Reference | - |
| Central | 214 (58.0) | 155 (42.0) | 369 | 1.2 (0.83-1.6) | 0.40 |
| Central East | 234 (56.7) | 179 (43.3) | 413 | 1.2 (0.89-1.7) | 0.23 |
| Southeast | 134 (58.8) | 94 (41.2) | 228 | 1.1 (0.78-1.59) | 0.56 |
| Champlain | 196 (61.2) | 124 (38.8) | 320 | 1.0 (0.72-1.4) | 0.99 |
| North Simcoe Muskoka | 90 (56.2) | 70 (43.8) | 160 | 1.2 (0.83-1.8) | 0.30 |
| Northeast | 125 (59.0) | 87 (41.0) | 212 | 1.1 (0.77-1.6) | 0.60 |
| Northwest | 46 (63.9) | 26 (36.1) | 72 | 0.90 (0.52-1.5) | 0.69 |
| Missing | 2 (66.7) | 1 (33.3) | 3 | - | - |

**Table 7. Number of separate drug prescriptions received by prostate cancer decedents.** The number of separate drug prescriptions received by each patient within the 90 days prior to study index date is represented, and stratified by whether the patient received any life prolonging therapy. The overall column represents the number and percent of the overall cohort in each drug prescription category.

| Number of Drugs Prescribed | No Life Prolonging Therapy  n (%)  (n=2132, 59.6%) | Life Prolonging Therapy  n (%)  (n=1443, 40.4%) | Overall  (n=3575) |
| --- | --- | --- | --- |
| 0 | 113 (75.8) | 36 (24.2) | 149 (4.2) |
| 1-2 | 112 (76.2) | 35 (23.8) | 147 (4.1) |
| 3-4 | 133 (65.5) | 70 (34.5) | 203 (5.7) |
| 5-6 | 224 (64.9) | 121 (35.1) | 345 (9.7) |
| 7+ | 1550 (56.8) | 1181 (43.2) | 2731 (76.3) |
